# Structural and biochemical analysis of human ADP-ribosyl-acceptor hydrolase 3 reveals the basis of metal selectivity and different roles for the two magnesium ions

**DOI:** 10.1016/j.jbc.2021.100692

**Published:** 2021-04-22

**Authors:** Yasin Pourfarjam, Zhijun Ma, Igor Kurinov, Joel Moss, In-Kwon Kim

**Affiliations:** 1 Department of Chemistry, University of Cincinnati, Cincinnati, Ohio, USA; 2 Department of Chemistry and Chemical Biology, NE-CAT APS, Cornell University, Argonne, Illinois, USA; 3 Pulmonary Branch, National Heart, Lung, and Blood Institute, National Institutes of Health, Bethesda, Maryland, USA

**Keywords:** ARH3, PARP1, ADP-ribosylation, hydrolysis, metal specificity, ADPR, ADP-ribose, ARH3, ADP-ribosyl-acceptor hydrolase 3, DBD, DNA-binding domain, HPF1, histone PARylation factor 1, ITC, isothermal titration calorimetry, MARylation, mono(ADP-ribosyl)ation, PAR, poly(ADP-ribose), PARG, PAR glycohydrolase, PARP, poly(ADP-ribose) polymerase, PARP1C, PARP1C catalytic domain, PARylation, poly(ADP-ribosyl)ation, PBST, PBS with Tween-20, PTM, post-translational modification

## Abstract

ADP-ribosylation is a reversible and site-specific post-translational modification that regulates a wide array of cellular signaling pathways. Regulation of ADP-ribosylation is vital for maintaining genomic integrity, and uncontrolled accumulation of poly(ADP-ribosyl)ation triggers a poly(ADP-ribose) (PAR)–dependent release of apoptosis-inducing factor from mitochondria, leading to cell death. ADP-ribosyl-acceptor hydrolase 3 (ARH3) cleaves PAR and mono(ADP-ribosyl)ation at serine following DNA damage. ARH3 is also a metalloenzyme with strong metal selectivity. While coordination of two magnesium ions (Mg^A^ and Mg^B^) significantly enhances its catalytic efficiency, calcium binding suppresses its function. However, how the coordination of different metal ions affects its catalysis has not been defined. Here, we report a new crystal structure of ARH3 complexed with its product ADP-ribose and calcium. This structure shows that calcium coordination significantly distorts the binuclear metal center of ARH3, which results in decreased binding affinity to ADP-ribose, and suboptimal substrate alignment, leading to impaired hydrolysis of PAR and mono(ADP-ribosyl)ated serines. Furthermore, combined structural and mutational analysis of the metal-coordinating acidic residues revealed that Mg^A^ is crucial for optimal substrate positioning for catalysis, whereas Mg^B^ plays a key role in substrate binding. Our collective data provide novel insights into the different roles of these metal ions and the basis of metal selectivity of ARH3 and contribute to understanding the dynamic regulation of cellular ADP-ribosylations during the DNA damage response.

Rapid and effective responses to extracellular and intracellular signals are crucial for the maintenance of genomic integrity and determination of cell fate (1). Post-translational modifications (PTMs) of proteins through a site-specific addition of chemical groups, such as phosphorylation, acetylation, and ADP-ribosylation, enable cells to dynamically regulate diverse biological pathways in an appropriate and timely manner (2). Poly(ADP-ribosyl)ation (PARylation) is a reversible PTM, in which the negatively charged ADP-ribose (ADPR) units are transferred from NAD^+^ to specific residues of target proteins, such as glutamate, aspartate, serine, arginine, and lysine (3, 4, 5). Poly(ADP-ribose) (PAR) polymerases (PARPs), particularly PARP1/ADP-ribosyltransferase diphtheria toxin-like 1, catalyze PARylation, generating a long chain of linear or branched polymers (6). PARylation plays a pleiotropic role in normal physiology as well as pathological conditions, including regulation of DNA damage repair (7), chromatin structure (8) and transcription (9), RNA metabolism (10), and cell fate determination (11).

However, uncontrolled accumulation of cellular PARylation is cytotoxic, leading to cell death (12). In mammals, cellular PAR levels are dynamically regulated by two PAR turnover enzymes, ADP-ribosyl-acceptor hydrolase 3 (ARH3) and PAR glycohydrolase (PARG) (13, 14). This turnover of PARylation is not only required for effective control of cellular signaling pathways but also restores the cellular energy reservoir (15, 16). Although both ARH3 and PARG cleave the α(1″-2′) *O*-glycosidic linkages in PAR substrates (15), there is a substantial difference in their structure and mechanism. The macrodomain-containing PARG has both endoglycohydrolase and exoglycohydrolase activity, generating protein-free oligo(ADP-ribose) chains and mono-ADP-ribose, respectively (17, 18, 19). PARG is unable to cleave terminal mono(ADP-ribosyl)ations attached to target proteins (20).

In contrast, ARH3 has a unique ARH fold and a di-Mg^2+^-containing catalytic center (21, 22, 23) and can efficiently reverse not only PARylation but also mono(ADP-ribosyl)ation (MARylation) at serine; ARH3 can also cleave *O*-acetyl-ADPR and α-NAD^+^ in a Mg^2+^-dependent manner releasing free ADPR (24, 25, 26). Notably, it has been shown that serine ADP-ribosylation, which is specifically synthesized by PARP1/histone PARylation factor 1 (HPF1) and PARP2/histone PARylation factor 2 complexes, is the major cellular PTM following DNA damage (27, 28, 29). Therefore, ARH3 plays an essential role in the complete reversal of DNA damage–induced cellular ADP-ribosylation. Consistent with this model, *ARH3*^−/−^ cells show enhanced accumulation of cellular PAR, leading to increased cell death following hydrogen peroxide–induced DNA damage (30). *ARH3* deficiency has been linked to the development of a progressive neurodegeneration phenotype (31, 32).

We and others recently reported structures of full-length or truncated ARH3 bound to Mg^2+^ and ADPR, its reaction product (21, 22, 23). We identified a structurally flexible Glu flap that undergoes a dramatic “closed-to-open” conformational transition upon ADPR binding that supports the specificity of ARH3 for the 1″-*O*-linkage in substrates (22). The Glu flap appears to function as a gate control for substrate entrance. Consistent with this structural plasticity of ARH3, two Mg^2+^ ions (Mg^A^ and Mg^B^) in the binuclear metal center exhibit different dynamics upon ligand binding (22), implying potentially different contributions of the two Mg^2+^ ions to ARH3 activity. Furthermore, it has been shown that Ca^2+^ significantly suppresses ARH3 activity, supporting the strong metal specificity of ARH3 (21, 22). However, despite the importance of metal coordination in ARH3 functions, the roles of each of the two Mg^2+^ ions and the basis of metal selectivity of ARH3 have not been defined.

Here, to address these previously unanswered questions on ARH3, we combined biochemical, biophysical, mutational, and structural tools. We found that Mg^2+^ coordination by ARH3 induces an approximately 60-fold increase in binding affinity to its product ADPR, whereas Ca^2+^ shows only a relatively moderate increase. Our new crystal structure of ARH3–ADPR–Ca^2+^ complex reveals that Ca^2+^ coordination significantly distorts the structure of the dimetallic catalytic center and interferes with optimal positioning of the 1″-OH group of the terminal ribose of ADPR, corresponding to the 1″-*O*-linkage in substrates, which results in impaired hydrolysis of PAR and serine mono(ADP-ribosyl)ated substrates. We further found that Mg^A^ and Mg^B^ play key but different roles for proper substrate alignment and substrate binding, respectively. Together, our results provide new insights into the metal-dependent mechanism and function of ARH3.

## Results

### Mg^2+^ coordination substantially enhances the ADPR-binding affinity of ARH3

**Figure 1:**
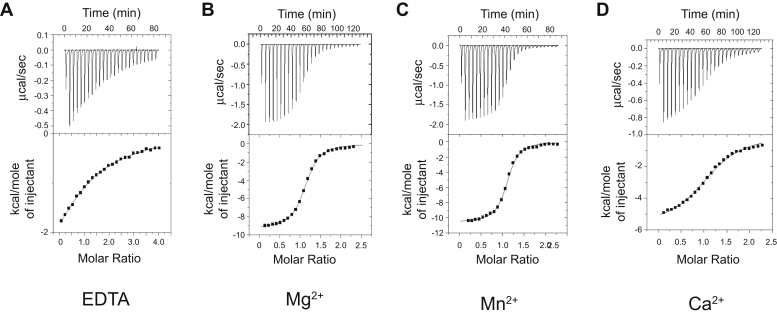
**Differential effects of metal coordination for substrate binding in ADP-ribosyl-acceptor hydrolase 3 (ARH3).** Isothermal calorimetry titration was used to measure ARH3 binding to ADP-ribose (ADPR) in the presence of EDTA (*A*), Mg^2+^ (*B*), Mn^2+^ (*C*), and Ca^2+^ (*D*). Binding affinities between ARH3 and ADPR were dramatically increased in the presence of Mg^2+^ and Mn^2+^ (*K*_*D*_ of 1.42 and 0.83 μM, respectively), compared with EDTA (*K*_*D*_ of 80.65 μM). The coordination of Ca^2+^ shows only a moderate increase in binding affinity (*K*_*D*_ of 7.81 μM). The ratio of ARH3:ADPR was approximately 1:1 in all experimental conditions. This is a representative assay of two independent experiments. The detailed thermodynamic parameters are described in Table 1.

A Mg^2+^-dependent catalytic mechanism and metal preference of ARH3 has been proposed by several groups (21, 22, 23, 33). In addition, it has been shown that Ca^2+^ effectively suppresses ARH3 functions, whereas Mn^2+^ shows minimal effects (21, 22). To gain insights into the basis for metal selectivity in ARH3, we used isothermal titration calorimetry (ITC) to determine the thermodynamic properties of ARH3 interactions with its reaction product ADPR in the absence (with EDTA) and the presence of Mg^2+^ and other divalent metals (Mn^2+^ and Ca^2+^) (Fig. 1, Table 1). The control injections into a buffer did not generate any significant heat (Fig. S1), indicating the signal comes from specific ADPR binding to ARH3. In all datasets, ARH3 binds to approximately one molecule of ADPR, which is consistent with reported crystal structures of ARH3–ADPR–Mg^2+^ complexes (21, 22, 23).

**Table 1:** ITC data of the interaction of ARH3^WT^, ARH3^D77A^, and ARH^D314A^ with ADPR and divalent metals

Proteins	Metals	*K*_*D*_ (μM)	N (sites)	ΔG (kcal M^−1^)	ΔH (kcal M^−1^)	−TΔS (kcal M^−1^)
ARH3^WT^	EDTA	80.65 ± 5.25	0.96 ± 0.08	−5.59	−5.164	−0.42
	Mg^2+^	1.42 ± 0.07	1.12 ± 0.01	−7.98	−9.29	1.31
	Mn^2+^	0.83 ± 0.05	1.09 ± 0.01	−8.29	−10.57	2.28
	Ca^2+^	7.81 ± 0.51	1.18 ± 0.01	−6.96	−5.71	−1.25
ARH3^D77A^	Mg^2+^	0.06 ± 0.01	1.18 ± 0.01	−9.87	−8.50	−1.37
ARH3^D314A^	Mg^2+^	62.89 ± 2.90	0.96 ± 0.04	−5.74	−10.36	4.61

The addition of Mg^2+^ to ARH3 increases its binding affinity to ADPR nearly 60-fold, in comparison to that of the metal-free enzyme state (in the presence of EDTA), *K*_*D*_^Mg^ of 1.42 μM *versus K*_*D*_^EDTA^ of 80.65 μM (Fig. 1, *A* and *B*, Table 1). The ADPR-binding affinity of ARH3 in the presence of Mn^2+^ is comparable to that with Mg^2+^ (*K*_*D*_^Mn^ of 0.83 μM), which is consistent with the similar level of PAR hydrolysis activity in the presence of Mg^2+^ or Mn^2+^ (Fig. 1*C*, Table 1) (22). In contrast, ARH3 showed a significantly lower ADPR-binding affinity in the presence of Ca^2+^ (*K*_*D*_^Ca^ of 7.81 μM), about a sixfold reduction in ADPR-binding affinity compared with that with Mg^2+^ (Fig. 1*D*, Table 1). Unlike Mg^2+^, the addition of Ca^2+^ resulted in less heat release (ΔH; −5.71 kcal M^−^ [Ca^2+^] *versus* −9.29 kcal M^−^ [Mg^2+^]) and an increase in entropy (TΔS) (Fig. 1, Table 1). The concentration of Ca^2+^ used for ITC (5 mM) is saturating, given that the Ca^2+^-dependent inhibitory was not further enhanced at higher Ca^2+^ concentrations (Fig. S2). Overall, the ADPR binding of ARH3 with inhibitory Ca^2+^ shows different isotherm and kinetic parameters from those with Mg^2+^ or Mn^2+^, which support enzymatic activity of ARH3, and shows features rather resembling those from metal-free ARH3 (in the presence of EDTA; Fig. 1*A*). Taken together, these results are consistent with the metal selectivity of ARH3 that enables its specific substrate recognition.

### Ca^2+^ binding distorts the binuclear metal center of ARH3

**Figure 2:**
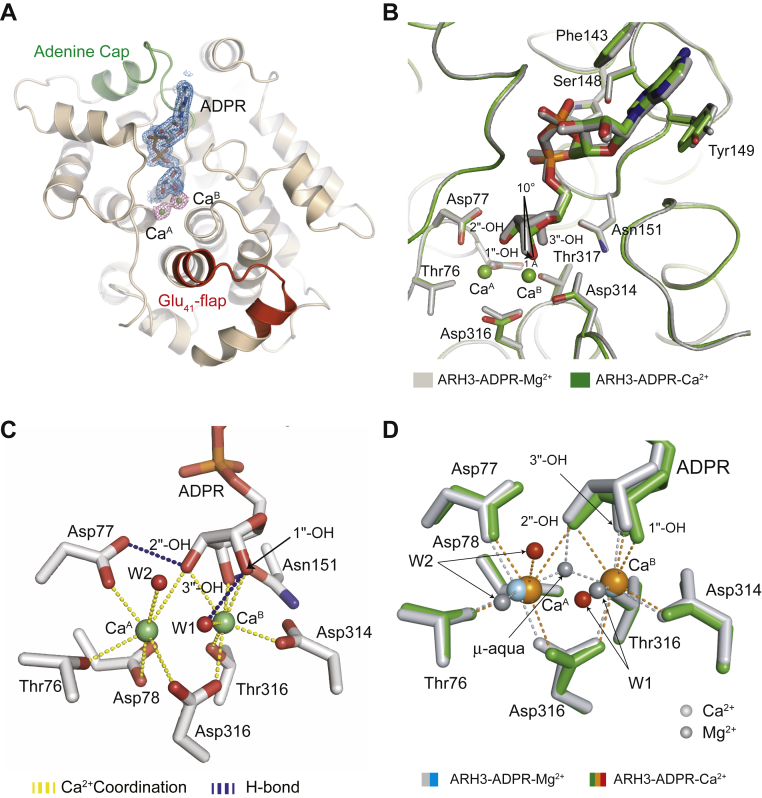
**The Ca**^**2+**^**coordination distorts the active-site architecture of ARH3.***A*, overall structure of ARH3^WT^–ADPR–Ca^2+^ with difference electron density maps (*F*_o_ − *F*_c_) for ADPR and Ca^2+^ ions contoured at 3.0 σ (*blue*: ADPR and *purple*: Ca^2+^). *B*, the structural superposition of ARH3^WT^–ADPR–Ca^2+^ (*green*) with ARH3^WT^–ADPR–Mg^2+^ (*gray*; Protein Data Bank ID: 6D36) reveals a tilted conformation of ADPR, in particular, the terminal ribose, in the Ca^2+^-bound form. *C*, a close-up view into the binuclear catalytic center and ADPR-binding mode in the Ca^2+^-bound form, which is distorted from those in the Mg^2+^-bound form (Fig. S3). Notably, the bridging water molecule (μ-aqua ligand) is missing, and all three hydroxyl groups of the terminal ribose interact with Ca^B^. *D*, an overlay of the active-site structures of ARH3^WT^–ADPR–Ca^2+^ (*green*) with ARH3^WT^–ADPR–Mg^2+^ (*gray*). Water ligands in Mg^2+^-bound form are colored *gray*, and those in Ca^2+^-bound form are colored *red*. ADPR, ADP-ribose; ARH3, ADP-ribosyl-acceptor hydrolase 3.

We previously reported the ligand-driven conformational switch in ARH3 that enables specific recognition and cleavage of the 1″-*O*-linkage in substrates (22). Furthermore, we showed that even a subtle distortion in the active-site architecture can have a detrimental effect on ARH3 functions. Since Ca^2+^ significantly suppresses the enzymatic activity of ARH3 (21, 22) and the ADPR binding in the Ca^2+^-bound form of ARH3 resembles the metal-free state of ARH3 (Fig. 1), we reasoned that Ca^2+^ binding likely distorts the active-site arrangement and metal-coordinating geometry in ARH3. To test this model and dissect the structural basis for the inhibitory effect of Ca^2+^, we determined the structure of full-length ARH3 in complex with ADPR and Ca^2+^ at a resolution of 1.75 Å (Fig. 2*A*, Table S2). Two metal ions (Ca^A^ and Ca^B^) and ADPR are positioned in a way similar to those found in the Mg^2+^-bound form (Fig. 2, *B* and *D*). However, the overall coordination geometry of the dimetallic catalytic center is remarkably different from that found in the Mg^2+^-bound form.

Unlike the Mg^2+^-bound form where both Mg^2+^ ions have a similar octahedral coordination geometry with six ligands, the coordination architecture of Ca^A^ and Ca^B^ shows a notable difference (Fig. 2, *C* and *D*, Table 2). Ca^B^ is coordinated with a total of seven ligands with an average metal–ligand distance of 2.49 Å (Fig. 2*C*, Table 2 and Table S1). This is slightly longer than that of Mg^B^ in the Mg^2+^-bound form (2.25 Å) and is likely because of the longer cationic diameter of calcium (radius of 0.99 Å) compared with magnesium (radius of 0.65 Å) (34). In contrast, Ca^A^ shows an octahedral but highly relaxed geometry. The average metal–ligand distance of Ca^A^ (2.82 Å) is substantially longer than that of Mg^A^ in the Mg^2+^-bound form (2.23 Å).

**Table 2:** Comparison of coordination of the two Ca^2+^ and Mg^2+^ (Protein Data Bank ID: 6D36) ions in the structures of ARH3–ADPR complexes

Metal	Distance (Å)	Coordinating atom		Distance (Å)	Coordinating atom
Ca^A^	2.7	Thr76-OG1	Mg^A^	2.4	Thr76-OG1
	2.4	Asp77-OD1		2.3	Asp77-OD1
	3.0	Asp78-OD2		2.3	Asp78-OD2
	2.6	Asp316-OD2		2.2	Asp316-OD2
	2.8	W2-O		2.2	W2-O
	3.0	ADPR (2″-OH)		2.0	μ-aqua-O
Ca^B^	2.4	Asp314-OD1	Mg^B^	2.2	As314-OD1
	3.0	Asp316-OD1		2.3	Asp316-OD1
	2.4	Thr317-OG1		2.3	Thr317-OG1
	2.5	W1-O		2.1	W1-O
	2.4	ADPR (1″-OH)		2.1	μ-aqua (μ-W)
	2.6	ADPR (2″-OH)		2.5	ADPR (3″-OH)
	2.4	ADPR (3″-OH)			

In the ARH3–ADPR–Ca^2+^ complex, the orientation of 1″-OH group of the terminal ribose, corresponding to the 1″-*O*-linkage in uncleaved substrates, is tilted toward Ca^B^ (Fig. 2*B*). With respect to the Mg^2+^-bound form, the 1″-OH group is rotated ∼10° in the Ca^2+^-bound form. This rotation induces a concomitant ∼1 Å displacement of 1″-OH toward Ca^B^. As a result, the 1″-OH group is directly coordinated to Ca^B^, whereas 2″-OH bridges the dimetallic center by interacting with both Ca^2+^ ions (Fig. 2*C*).

Notably, the metal-bridging water (μ-aqua) is missing in the Ca^2+^-bound form (Fig. 2, *C* and *D*). In the Mg^2+^-bound form, this μ-aqua ligand simultaneously engages both Mg^2+^ ions and the 2″-OH. This μ-aqua ligand is missing in three of four ARH3–ADPR–Ca^2+^ complexes in the asymmetric unit, and only a very weak electron density was found in the last ARH3 molecule. Instead, two Ca^2+^ ions are bridged by 2″-OH of the terminal ribose (Fig. 2*C*), which causes the tilted confirmation of the terminal ribose. This absence of the bridging water ligand may explain the shorter metal–metal distance in the Ca^2+^-bound form than in the Mg^2+^-bound form (3.1 Å [Ca^2+^–Ca^2+^] *versus* 3.3 Å [Mg^2+^–Mg^2+^]) (Table S1).

Taken together, our findings suggest that the distorted di-Ca^2+^ metal center and the tilted conformation of 1″-OH, a position for the scissile *O*-linkage in substrates, are likely to interfere with a precise alignment of substrates for nucleophilic attack, resulting in suppression of enzymatic activities of ARH3. These results also support the hypothesis that a subtle change in the metal-coordination arrangement can result in a detrimental effect on ARH3 functions.

### Metal-coordinating acidic residues are critical for the catalytic activity of ARH3

**Figure 3:**
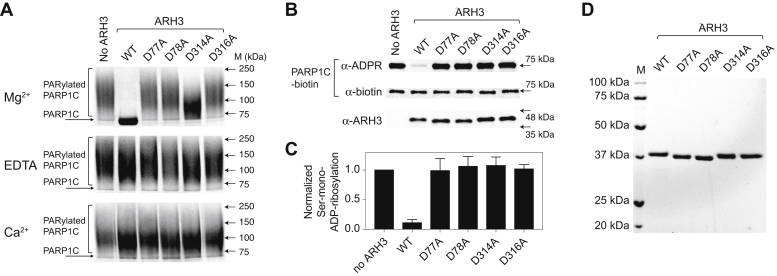
**Metal-coordinating acidic residues are critical for ARH3 functions.***A*, effects of metal coordination on PAR hydrolytic activity. WT and metal-coordination mutants of ARH3 (D77A, D78A, D314A, and D316A) were incubated with PARylated PARP1C substrates to measure PAR hydrolytic activity in the presence of EDTA, Mg^2+^, and Ca^2+^. With WT ARH3, Mg^2+^ is required for PAR hydrolysis, whereas EDTA and Ca^2+^ inhibit, which is consistent with our previous report (22). All ARH3 mutants show impaired PAR hydrolytic activity. *B*, the serine MARylated PARP1C was treated with 60 nM concentration of WT ARH3 and metal-coordination mutants of ARH3 in the presence of Mg^2+^ (5 mM). While ARH3^WT^ efficiently cleaves ADP-ribose–serine, all metal-coordination mutants were inactive, supporting the hypothesis that the metal coordination is critical for ARH3 functions. *C*, quantification of the ADP-ribose–serine hydrolytic activity is shown in panel *B*. The data are shown with mean values and standard deviations from three independent experiments. *D*, a gel image showing the purified WT ARH3 and ARH3 mutants used in this study. ARH3, ADP-ribosyl-acceptor hydrolase 3; MAR, mono(ADP-ribose); PAR, poly(ADP-ribose); PARP1C, PARP1C catalytic domain.

To gain further insights into the structure–function relationship of the dimetallic catalytic center and its metal-coordination geometry, we substituted acidic residues that directly coordinate metals (Asp77, Asp78, Asp314, and Asp316) with alanine. Asp77 and Asp314 interact with both ADPR and Mg^2+^, whereas Asp78 and Asp316 coordinate only Mg^A^ and Mg^B^, respectively but do not directly interact with ADPR. We purified these mutants (Fig. 3*D*) and monitored their PAR and serine MARylation hydrolytic activities in the presence of Mg^2+^, EDTA, and Ca^2+^ (Fig. 3).

The addition of a metal-chelating EDTA or Ca^2+^ effectively suppresses PAR hydrolysis by ARH3, which is in good agreement with published results (Fig. 3*A*) (21, 22). Among ARH3 mutants, only ARH3^D314A^ showed some residual PAR hydrolysis activity in the presence of Mg^2+^, and the others were nearly inactive in all three tested metal conditions (Fig. 3*A*).

While the WT enzyme efficiently reversed the serine-linked MARylated PARP1 substrate, all tested ARH3 mutants lacked enzymatic activity (Fig. 3, *B* and *C*). Collectively, these results suggest that metal-coordinating residues in the active site of ARH3 are critical for both PAR and MAR hydrolysis, and de-MARylation function of ARH3 appears to require a more strict structural integrity to specifically recognize and/or cleave MARylated substrates.

### Mg^A^ is required for precise substrate alignment, whereas Mg^B^ is important for substrate binding

In WT ARH3, Asp77 not only interacts with 2″-OH of the terminal ribose but also coordinates Mg^A^. Thus, Asp77 appears to be important for the maintenance of integrity of Mg^A^ and the optimal orientation of the terminal ribose for catalysis. Similarly, on the other side of the active site, Asp314 interacts with 3″-OH of the terminal ribose and Mg^B^. Asp314 seems to contribute to the integrity of Mg^B^, given that the substitution of Asp314 with glutamate eliminates Mg^B^ from the active site (22). To gain further structural basis for different alterations in enzymatic activity in D77A and D314A mutants and to better understand the roles of each Mg^2+^ ion in ARH3, we determined the high-resolution crystal structures of ARH3^D77A^ and ARH3^D314A^ bound to ADPR and Mg^2+^ at a resolution of 1.85 and 1.80 Å, respectively.

**Figure 4:**
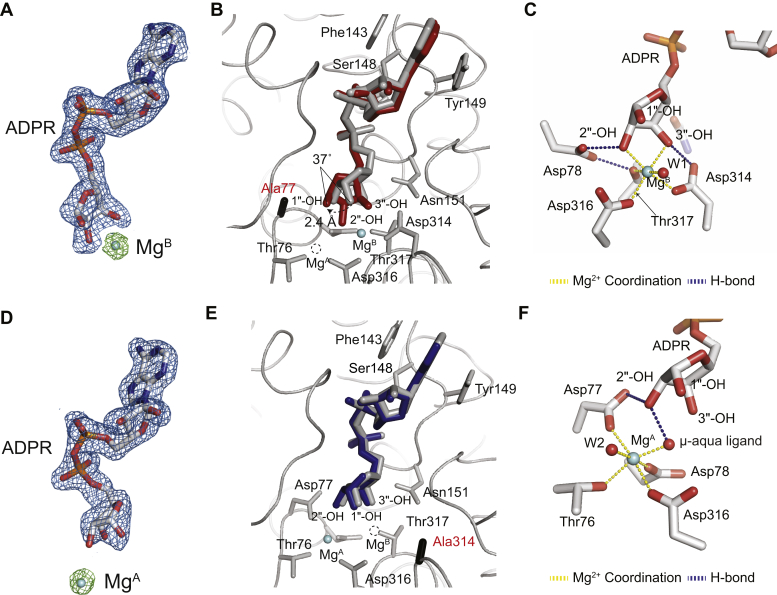
**Structural comparison reveals different roles of Mg**^**A**^**and Mg**^**B**^**in the ARH3 active site.***A*, difference electron density maps (*F*_o_ − *F*_c_) for ADPR and Mg^2+^ ions contoured at 3.0 σ (*blue*: ADPR and *green*: Mg^2+^). *B*, structural overlay of ARH3^D77A^–ADPR–Mg^2+^ (*red*) and ARH3^WT^–ADPR–Mg^2+^ (*gray*). The substitution of Asp77 to Ala results in missing Mg^A^, a dramatically rotated terminal ribose (∼37°), and a 2.4 Å displacement of 1″-OH, with respect to the WT ARH3. This structural rearrangement would likely interfere with correct positioning of substrate for catalysis, supporting the role of Mg^A^ in the precise substrate alignment. The position of the missing Mg^A^ is shown with a *dotted circle*. *C*, a close-up view of the active site and ADPR-binding mode in ARH3^D77A^. *D*, difference electron density maps (*F*_o_ − *F*_c_) for ADPR and Mg^2+^ ions contoured at 3.0 σ (*blue*: ADPR and *green*: Mg^2+^). *E*, structural overlay of ARH3^D314A^–ADPR–Mg^2+^ (*blue*) and ARH3^WT^–ADPR–Mg^2+^ (*gray*). The substitution of Asp314 with Ala results in missing Mg^B^, whereas the overall ADPR-binding mode is not changed compared with ARH3^WT^. This structure, along with the dramatically reduced ADPR-binding affinity of ARH3^D314A^ (Fig. 5, Table 1), suggests that Mg^B^ plays a key role in substrate binding. The position of the missing Mg^B^ is shown with a *dotted circle*. *F*, a close-up view of the active site and ADPR-binding mode in ARH3^D314A^. ADPR, ADP-ribose; ARH3, ADP-ribosyl-acceptor hydrolase 3.

**Figure 5:**
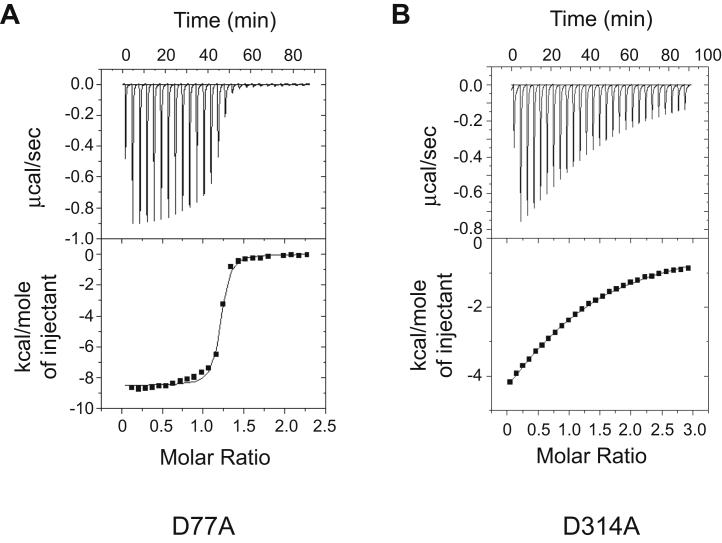
**Differential effects of the substitution of metal-coordinating residues in substrate binding.***A*, ADPR binds to ARH3^D77A^ with a significantly higher affinity (*K*_*D*_ of 0.06 μM) compared with ARH3^WT^ (1.42 μM). *B*, in contrast, ARH3^D314A^ shows a substantially weaker binding affinity for ADPR (*K*_*D*_ of 62.89 μM). This is the representative assay of two independent experiments. The detailed thermodynamic parameters are described in Table 1. ADPR, ADP-ribose; ARH3, ADP-ribosyl-acceptor hydrolase 3.

Structural analysis of ARH3^D77A^ reveals that Mg^A^ is missing (Fig. 4, *A* and *B*). This lack of Mg^A^ causes a large-scale rearrangement in the active site. First, the terminal ribose is rotated ∼37° using Mg^B^ as a pivot (Fig. 4*B*). Because of this rotation, which is an even greater degree of rotation than that found in the Ca^2+^-bound form of ARH3 (Fig. 2), the 2″-OH group replaces the metal-bridging μ-aqua ligand and directly interacts with Mg^B^. In this conformation, the 2″-OH group makes additional hydrogen bonds with side chains of Asp78 and Asp316, and the 1″-OH group makes a new hydrogen bond with the main carbonyl chain of Gly115 (Fig. 4*C* and Fig. S2). Consistently, ARH3^D77A^ shows a 23-fold increase in ADPR-binding affinity, compared with ARH3^WT^ (Fig. 5*A*, Table 1). As a result, the 1″-OH group is displaced ∼2.4 Å toward Ala77 (Fig. 4*B*). This significantly rotated conformation of ADPR in ARH3^D77A^, with respect to ARH3^WT^, is likely to interfere with a correct substrate positioning for catalysis (Fig. 4*B*). Together, the coordination of Mg^A^ appears to contribute to the optimal alignment of substrate, while it sacrifices the overall substrate-binding affinity of ARH3.

In the structure of ARH3^D314A^, similar to ARH3^D314E^ (22), Mg^B^ is missing (Fig. 4*D*). Unlike ARH3^D77A^, the orientation of ADPR in ARH3^D314A^ is nearly identical to that in ARH3^WT^, and critical residues for the formation of the binuclear metal center occupy nearly identical positions (Fig. 4*E*). This finding is in line with the proposed important role of Mg^A^ for precise substrate alignment. The geometry of Mg^A^ is also maintained in an octahedral shape as seen in ARH3^WT^ (Fig. 4*F*). Overall, in contrast to ARH3^D77A^, ARH3^D314A^ appears to show a limited effect on the alignment of the terminal ribose (Fig. 4*F* and Fig. S2). Rather, Mg^B^ appears to be important for substrate binding. The 3″-OH group of the terminal ribose, which is originally in direct coordination with Mg^B^ and the side chain of Asp314 (Fig. S3), lacks interactions with the enzyme in the structure of ARH3^D314A^ and is disordered (Fig. 4*F*). Consistent with these findings, ARH3^D314A^ shows a 44-fold decrease in ADPR-binding affinity compared with ARH3^WT^ (Fig. 5*B*, Table 1). Notably, this reduced ADPR-binding affinity mimics the metal-free state of ARH3 (Fig. 1, Table 1), further supporting the hypothesis that Mg^B^ is critical for substrate binding in ARH3.

Taken together, these findings imply that Mg^A^ is important for the optimal positioning of substrates, whereas Mg^B^ plays a key role for substrate binding in ARH3.

## Discussion

Our combined structural, biochemical, and mutational analysis reveal new insights into the metal selectivity and different roles of two metal ions in ARH3. Our new crystal structure of the ARH3–ADPR–Ca^2+^ complex explains the strong preference of Mg^2+^ over Ca^2+^ and the Ca^2+^-mediated inhibition of ADP-ribosyl-acceptor hydrolase activities of ARH3. Although overall folding of ARH3 remains nearly identical upon substitution of Mg^2+^ with Ca^2+^ (Fig. 2*B*), our careful structural analysis showed that Ca^2+^ coordination in ARH3 results in significant rearrangements in geometry, coordination, and metal–ligand distances of the binuclear metal center, explaining the impaired ARH3 activities in the presence of Ca^2+^ (Fig. 3).

In contrast to Mg^2+^ (Fig. 2*D*, Table 2), Ca^2+^ ions can coordinate up to eight metal ligands (35, 36). In our structure, Ca^B^ is coordinated with seven ligands, including the 1″-OH (scissile *O*-linkage in substrates) and 2″-OH, and Ca^A^ shows a very relaxed octahedral geometry (Fig. 2*C*). The significantly distorted active-site structure and reduced binding affinity in the Ca^2+^-bound form of ARH3 are consistent with the proposed roles of Mg^A^ and Mg^B^ in substrate alignment and binding. For example, the highly relaxed Ca^A^ geometry, compared with the Mg^2+^-bound form, likely interferes with an optimal substrate alignment in ARH3 and following nucleophilic attack on the distal ribose. In addition, Ca^2+^ ions can reduce the chemical reactivity of metal-bound water molecules. Previous studies reported that a Ca^2+^-bound water molecule has a higher p*K*a (12.9) in comparison to that bound to Mg^2+^ (11.4) or Mn^2+^ (10.9) (34). Consequently, a nucleophilic attack on substrates becomes less favorable (37). Finally, it is possible that the larger size of Ca^2+^ (radius of 0.99 Å) (34), compared with Mg^2+^ (radius of 0.65 Å) and Mn^2+^ (radius of 0.80 Å), along with its preference for a higher coordination number (up to eight ligands), might cause steric interference with the coordinated oxygen atoms, possibly perturbing the stability of the transition state.

Our atomic-resolution structures and mutational analysis define the different roles of two Mg^2+^ ions in ARH3. Although several mutations were previously reported (38), our results provide extended and atomic resolution insights into the roles of key acidic residues in the binuclear catalytic center. Furthermore, we compared effects of mutations both on serine MARylated substrates and PARylated substrates. In the active site of ARH3, acidic residues, including Asp77, Asp78, Asp314, and Asp316, maintain the integrity of the bimetallic center and render the ideal alignment of the substrate for catalysis (22). These metal-coordinating acidic residues are critical for hydrolysis of PAR and ADPR–serine (Fig. 3). In the ARH3^D77A^ structure, in which Mg^A^ is missing, the terminal ribose of ADPR is rotated ∼37°, which is approximately four times greater than the rotation found in the Ca^2+^-bound form (Figs. 2 and 4). This distorted conformation, together with a surprisingly increased binding affinity to ADPR in ARH3^D77A^ (Table 1), would presumably interfere with correct substrate positioning for catalysis (Fig. 4*B*). Given the missing Mg^A^, a complete loss of enzymatic activity, and an increased ADPR-binding affinity in ARH3^D77A^ (Fig. 3*A*), Mg^A^ appears to play an important role in catalysis by sacrificing the substrate-binding affinity. Also, these findings raise the possibility that Asp77 may function as a catalytic residue. In contrast, ARH3^D314A^, in which Mg^B^ is missing, shows nearly identical active-site structure, but an impaired ADPR-binding affinity, which is comparable to the metal-free ARH3 (Table 1). The limited alteration in the ADPR-binding mode in the absence of Mg^B^ further supports the conclusion that Mg^A^ is crucial for substrate alignment. Taken together, we propose that the coordination of Mg^A^ appears to contribute to the optimal alignment of substrate, whereas Mg^B^ is crucial for substrate binding.

Among the ARH–macrodomain family of ADP-ribosylation reversal enzymes, ARH3 is a unique multitasking metalloenzyme that regulates cellular concentrations of multiple ADP-ribosylated substrates, including PAR (Fig. 3*A*), serine MAR (Fig. 3*B*), *O*-acetyl-ADPR (26, 39), and α-NAD^+^ (25). These ADP-ribosylated substrates play key roles in cellular signaling pathways, such as the DNA damage response and determination of cell death. Although more work needs to be done to define the comprehensive metal-dependent catalytic mechanism in ARH3, the structural and biochemical data presented here provide detailed insights into the dynamic active-site rearrangement upon coordination of different divalent metals and suggest specific and different roles of Mg^A^ and Mg^B^. The observed metal preference of ARH3 to Mg^2+^ supports the structural plasticity and broad substrate specificity of ARH3, while maintaining the structural integrity of the active site. Finally, our data suggest that the change in cellular concentrations of divalent metals, such as Ca^2+^, can modulate ARH3 functions.

## Experimental procedures

### Plasmids and protein purification

WT ARH3 (ARH3^WT^), the DNA-binding domain (DBD; residues 1–374), and the PARP1C catalytic domain (PARP1C; residues 375–1014) of human PARP1 were purified as described previously (22). To purify biotinylated PARP1C, the corresponding gene was cloned into a pET28a vector with a C-terminal biotin affinity peptide tag for biotinylation and an N-terminal His6 tag for purification. PARP1C was coexpressed in *Escherichia coli* BL21 cells expressing the biotin ligase BirA that biotinylates the biotin affinity peptide tag on its lysine residue. Genes for mutants of ARH3 were synthesized and cloned into a modified pET21b vector with an N-terminal His6 tag and a following cleavage site for PreScission protease (pET21b-His6-pps) by Gene Universal Inc. All plasmids were sequenced, and mutations were confirmed. The biotinylated PARP1C and ARH3 mutant proteins were purified using the same protocol as ARH3^WT^. A gene for human HPF1 was synthesized and cloned into the pET21b-His6-pps by Gene Universal Inc. HPF1 was purified as described previously (29).

### ITC

ITC experiments were performed at 25 °C in a buffer containing 150 mM NaCl, 100 mM Tris at pH 7.5, and 5 mM divalent metals (MgCl_2_, MnCl_2_, and CaCl_2_) or EDTA (Sigma–Aldrich). The WT or mutants of ARH3 were placed in a cell at 50 μM concentration, and ADPR was placed in the injection syringe at 660 μM concentration. Overall, 26 injections (10 μl per injection except for the first sample [5 μl]) were administered with an interval of 5 min between peaks to allow the baseline to be stabilized. Control experiments were conducted under the same condition to determine the heat of dilution by injecting ADPR to buffers, which shows no significant heat generation (Fig. S1). The obtained plots were fitted with a single-site binding model in the Origin software package (MicroCal, Inc).

### PAR turnover assay

A gel-based PAR-turnover assay was performed to evaluate the enzymatic activity of ARH3^WT^, ARH3^D77A^, ARH3^D78A^, ARH3^D314A^, and ARH3^D316A^, as described previously (14, 40). Briefly, 2 μM of the catalytic domain of human PARP1 (PARP1C; residues 375–1014) was auto-PARylated at 37 °C for 30 min in the presence of 2 μM DBD of PARP1 (residues 1–374), 2 μM double-stranded DNA, and 400 μM β-NAD^+^ (Sigma) in a buffer containing 100 mM NaCl, 50 mM Tris (pH 7.5), 10 mM MgCl_2_, and 2 mM DTT. ARH3 proteins (in the presence of 5 mM EDTA, Mg^2+^, or Ca^2+^) with final concentrations of 2 μM were mixed with the PARylated PARP1C substrate, followed by incubation for 1 h in 37 °C. Reactions then were stopped by adding 4× SDS-loading dye (Bio-Rad) and visualized by Coomassie blue staining of SDS-polyacrylamide gels.

### Serine mono(ADP-ribosyl)ation turnover assay

To generate serine-linked MARylated substrate, 4 μM of PARP1C was first PARylated in the presence of 4 μM PARP1 DBD, 8 μM HPF1, 2 μM double-stranded DNA, and 400 μM β-NAD^+^ (Sigma–Aldrich) in a buffer containing 100 mM NaCl, 50 mM Tris (pH 7.5), 10 mM MgCl_2_, and 2 mM DTT. The reaction then was passed through a PD-10 column to remove excess β-NAD^+^. PARP inhibitor, Olaparib (SelleckChem), at 8 μM was then added before performing dePARylation of the substrate by incubating with 10 nM PARG for 30 min in 37 °C. The generated serine-linked MARylated PARP1C was passed through a PD-10 column to remove protein-free PAR and ADPR units, followed by aliquoting and storing in −80 °C. ARH3 proteins at a final concentration of 60 nM were treated into the serine-MARylated substrate in a buffer containing 50 mM Tris–HCl (pH 7.5), 50 mM NaCl, and 5 mM MgCl_2_, followed by incubation for 1 h in 37 °C. Reactions then were stopped by adding 4× SDS-loading dye and resolved by 10% SDS-polyacrylamide gels.

### Western blotting

To visualize and quantify the level of serine-MARylation, we performed Western blotting on samples. Reactions were transferred to polyvinylidene fluoride membranes. Polyvinylidene fluoride membranes first were blocked by incubation in PBS with Tween-20 (PBST) buffer containing 5% nonfat skim milk for 1 h at room temperature. After one washing step with PBST, membranes were incubated with anti–pan-ADPR antibody (Sigma–Aldrich; 3 μg/ml final concentration), anti-ARH3 N-terminal antibody (Aviva System Biology) (1/1000 dilution), and antibiotin antibody (Invitrogen; 0.25 μg/ml final concentration) overnight at 4 °C. After three steps of wash with PBST, blots were incubated with horseradish peroxidase–conjugated polyclonal secondary anti-rabbit antibody (Thermo Fisher; 1/10,000 dilution) for 1 h at room temperature. Afterward, membranes were washed three times with PBST and finally visualized by enhanced chemiluminescence detection kit using the iBRIGHT-FL1000 imager (Thermo Fisher). Bands were quantified using ImageJ software (National Institutes of Health).

### Crystallization and data collection

The ARH3^WT^, ARH3^D77A^, and ARH3^D314A^ (10 mg/ml) were cocrystallized with 5 mM ADPR (Sigma–Aldrich) and 5 mM Ca^2+^ (for ARH3^WT^) or Mg^2+^ (for ARH3^D77A^ and ARH3^D314A^) by hanging-drop vapor diffusion (22). Briefly, 1 μl of protein samples was mixed with 1 μl mother liquor containing 0.1 M sodium acetate buffer (pH 4.5), 0.1 M MgCl_2_ or CaCl_2_, and 20 to 24% PEG 4000. About 24-well plates were placed in 22 °C incubator until crystals appeared. Harvested crystals were transferred into a cryoprotectant solution (26% PEG 4000; 0.1 M sodium acetate; pH 4.5; 0.1 M corresponding divalent metal; 5 mM ADPR; and 10% glycerol) before flash cooling in liquid nitrogen for data collection.

X-ray diffraction data were collected at the NE-CAT 24ID-E beamline at the Advanced Photon Source. The ARH3^WT^–ADPR–Ca^2+^ complex crystals (P1; four ARH3–ADPR–Ca^2+^ complexes per asymmetric unit) diffracted to a resolution of 1.75 Å, ARH3^D77A^–ADPR–Mg^2+^ complex crystals (P1) diffracted to a resolution of 1.85 Å, and ARH3^D314A^–ADPR–Mg^2+^ complex crystals (P1) diffracted to a resolution of 1.80 Å. X-ray datasets were collected with an Eiger 16M detector (Dectris) and processed using HKL2000 (41) and SCALEPACK (HKL Research, Inc.) (41, 42). Data collection statistics are shown in Table S2.

### Structure determination

Structures were determined by molecular replacement using MOLREP (43, 44) in the CCP4 suite (44) with the ARH3^WT^–ADPR–Mg^2+^ structure (Protein Data Bank ID: 6D36) as a search model. Models were manually built using Coot (45) and refined in PHENIX (46); *R*_factor_ of 14.9% and *R*_free_ of 18.8% for ARH3^WT^–ADPR–Ca^2+^, *R*_factor_ of 18.1% and *R*_free_ of 22.7% for ARH3^D77A^–ADPR–Mg^2+^, and *R*_factor_ of 18.8% and *R*_free_ of 22.7% for ARH3^D314A^–ADPR–Mg^2+^. Crystallographic data are shown in Table S2. Ramachandran plots indicate that >98% of residues in all three structures are in favored regions, and all others are in allowed regions. No outlier residue is observed.

## Data availability

Atomic coordinates and structure factors of the ARH3^WT^–ADPR–Ca^2+^, ARH3^D77A^–ADPR–Mg^2+^, and ARH3^D314A^–ADPR–Mg^2+^ complexes have been deposited in the Protein Data Bank under accession numbers 7L9F, 7L9H, and 7L9I, respectively.

## Supporting information

This article contains supporting information (22).

## Conflict of interest

The authors declare that they have no conflicts of interest with the contents of this article.
