# Supplementary material for: Structural and biochemical analysis of human ADP-ribosyl-acceptor hydrolase 3 reveals the basis of metal selectivity and different roles for the two magnesium ions
Source: J Biol Chem. 2021 Apr 22;296:100692. doi: 10.1016/j.jbc.2021.100692 (PMC8141533; doi:10.1016/j.jbc.2021.100692)
Supplement: Supplemental Figures S1–S3 and Tables S1 and S2 [file mmc1.pdf]

Supporting Information

Structural and biochemical analysis of human ADP-ribosyl-acceptor hydrolase 3 (ARH3) reveals the basis of metal selectivity and different roles for the two Mg ions

Yasin Pourfarjam[1], Zhijun Ma[1], Igor Kurinov[2], Joel Moss[3], and In-Kwon Kim[1,*]

From the [1] Department of Chemistry, University of Cincinnati, 301 Clifton Ct, Cincinnati, OH 45221, USA; [2] Cornell University, Department of Chemistry and Chemical Biology, NE-CAT APS, Building 436E, 9700 S. Cass Ave., Argonne, IL 60439; [3] Pulmonary Branch, National Heart, Lung, and Blood Institute, National Institutes of Health, Bethesda, MD 20892

Running title: *Different roles of two metal ions in ARH3*

* To whom correspondence should be addressed: In-Kwon Kim, Department of Chemistry, University of Cincinnati, 301 Clifton Ct, Cincinnati, OH 45221, USA; E-mail: kimiw@ucmail.uc.edu; Tel.: (513) 556-1909; Fax.: (513) 556-9239.

Supporting information contents:

Figure S1. Heat of ADPR injection into the buffer.

Figure S2. Ca^{2+}-dependent inhibition of ARH3 activity.

Figure S3. A close-up view into the active site of the $ARH3^{WT}$–ADPR–Mg^{2+} complex.

Table S1. Calcium and Magnesium coordination parameters.

Table S2. Crystallographic data statistics.

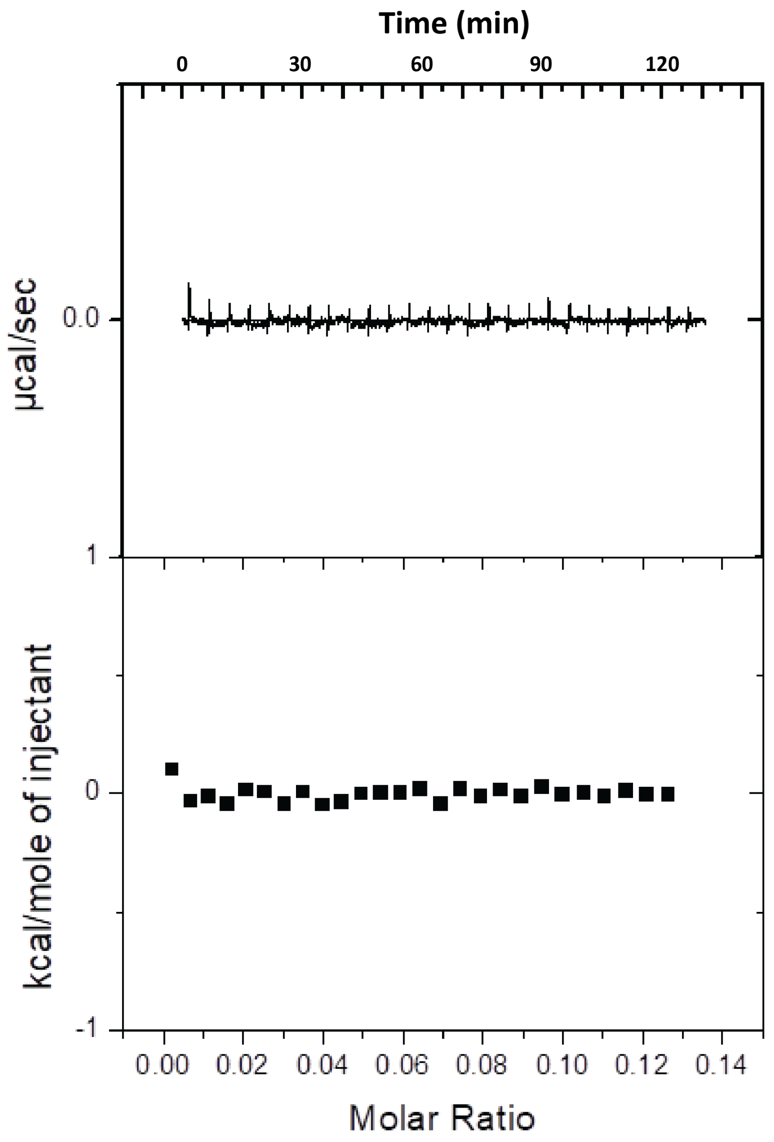

Figure S1. Heat of ADPR injection into the buffer. ADP-ribose (660 µM) in syringe was injected into the cell containing a buffer (100 mM Tris pH 7.5, 150 mM NaCl, and 5 mM $MgCl_2$).

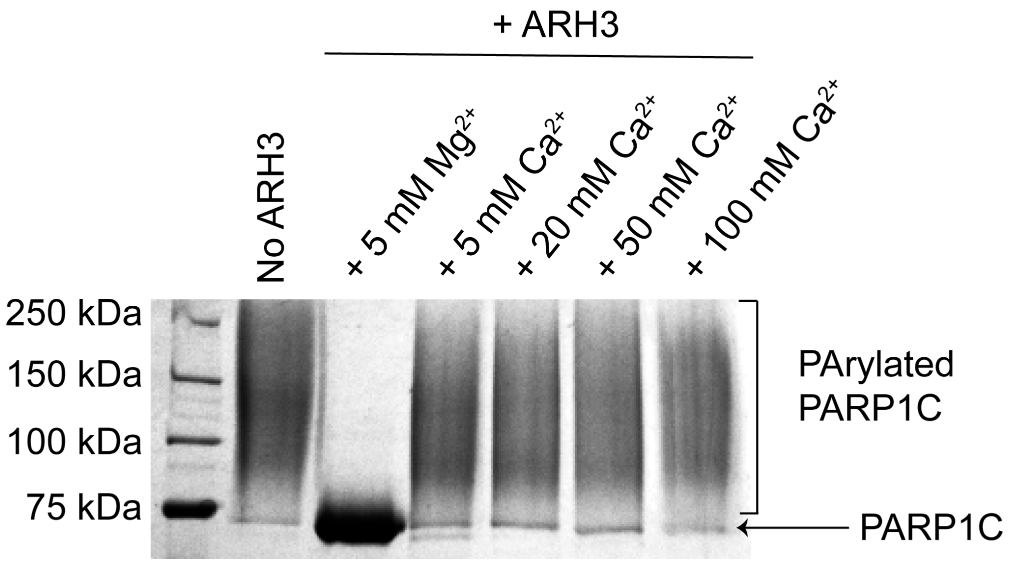

Figure S2. Ca^{2+}-dependent inhibition of ARH3 activity. The PAR hydrolysis activity of ARH3 was monitored in the presence of Mg^{2+} and increasing concentrations of Ca^{2+}. 5 mM Ca^{2+} was sufficient to effectively inhibit ARH3 activity and was used for ITC experiments.

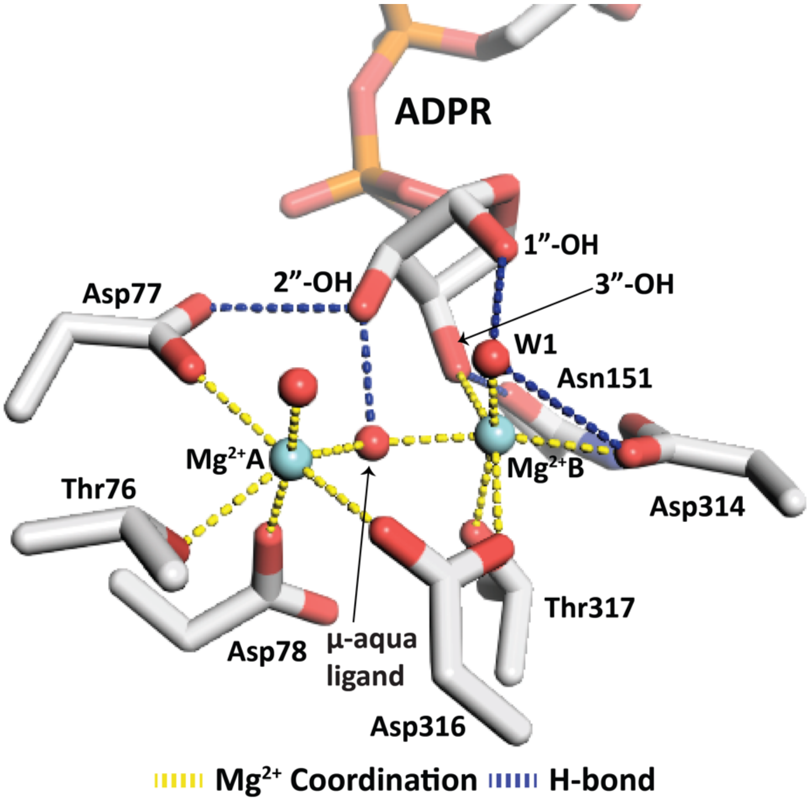

Figure S3. A close-up view into the active site of ARH3^{WT}–ADPR–Mg^{2+} structure (1). Mg^A and Mg^B are bridged by a water molecule (μ aqua ligand), which is expelled upon substitution of magnesium cations by calcium. In addition, in contrast to Ca^{2+}-bound structure in which Ca^B coordinates all three hydroxyl groups of the terminal ribose″ (1″-OH, 2″-OH, 3″-OH), Mg^B is in direct interaction with only the 3″-OH group.

ARH3–ADPR–Ca^{2+}		ARH3–ADPR–Mg^{2+}	
Ca^{2+} diameter (Å)	1.06	Mg^{2+} diameter (Å)	0.72
Ca^{A}-Ligands number	6	Mg^{A}-Ligands number	6
Ca^{B}-Ligands number	7	Mg^{B}-Ligands number	6
Ca^{A}-Ligands distance (Å, Ave)	2.23	Mg^{A}-Ligands distance (Å, Ave)	2.49
Ca^{B}-Ligands distance (Å, Ave)	2.25	Mg^{B}-Ligands distance (Å, Ave)	2.82
Ca^{A}-Ca^{B} distance (Å, Ave)	3.3	Mg^{A}-Mg^{B} distance (Å, Ave)	3.1

Table S1. Comparision of coordination parameters between ARH3–ADPR–Ca^{2+} and ARH3–ADPR–Mg^{2+} complexes.

Table S2. Crystallographic data statistics. *Values in parentheses are for highest-resolution shell. Each dataset was collected from a single crystal.

	*h*ARH3^{WT}-ADPR-Ca^{2+}	*h*ARH3^{D77A}-ADPR-Mg^{2+}	*h*ARH3^{D314A}-ADPR-Mg^{2+}
**Data collection**			
Space group	P1	P1	P1
Cell dimensions			
a, b, c (Å)	44.9, 71.4, 115.6	44,7, 71.6, 115.9	44.8, 71.4, 115.8
α, β, γ (°)	93.9, 96.3, 107.1	94.2, 94.6, 107.6	94.0, 94.6, 107.8
Wavelength (Å)	0.97	0.97	0.97
Resolution (Å)	67.88 - 1.75	61.29 - 1.85	67.64 – 1.8
R_{sym} (%)	2.8 (11.7)	7.8 (29.2)	12.1 (55.5)
$I / \sigma I$	20.0 (6.6)	8.3 (2.7)	4.6 (1.2)
Completeness (%)	91.5 (90.3)	89.5 (91.0)	89.8 (87.7)
Redundancy	2.2 (2.2)	1.9 (1.9)	1.9 (1.8)
**Refinement**			
Resolution (Å)	67.88 - 1.75	61.29 – 1.85	67.64 – 1.80
No. reflections	125,690	103,770	112,760
R_{work} / R_{free}	14.9/18.8	18.1/22.7	18.8/22.7
No. atoms			
Protein	10014	9845	9986
Ligand/ion	152	148	148
Water	1214	909	857
B-factors			
Protein	20.4	22.1	22.3
Ligand/ion	21.6	20.2	24.5
Water	60.6	30.1	28.8
R.m.s deviations			
Bond lengths (Å)	0.009	0.012	0.006
Bond angles (°)	0.97	1.14	0.76

REFERENCES

1. Pourfarjam, Y., Ventura, J., Kurinov, I., Cho, A., Moss, J., and Kim, I. K. (2018) Structure of human ADP-ribosyl-acceptor hydrolase 3 bound to ADP-ribose reveals a conformational switch that enables specific substrate recognition. *J Biol Chem* **293**, 12350-12359
